# Prognostic Impact of Percutaneous Coronary Intervention in Chronic Dialysis Patients with Acute Myocardial Infarction: *Findings from the Lombardy Health Database*

**DOI:** 10.31083/j.rcm2405135

**Published:** 2023-04-28

**Authors:** Nicola Cosentino, Simonetta Genovesi, Alice Bonomi, Filippo Trombara, Monica Ludergnani, Olivia Leoni, Francesco Bortolan, Piergiuseppe Agostoni, Giancarlo Marenzi

**Affiliations:** ^1^Centro Cardiologico Monzino, IRCCS, 20138 Milan, Italy; ^2^School of Medicine and Surgery, Nephrology Clinic, Milano-Bicocca University, 20900 Monza, Italy; ^3^Istituto Auxologico Italiano, IRCCS, 20133 Milan, Italy; ^4^Regional Epidemiological Observatory, Lombardy Region, 20100 Milan, Italy; ^5^Cardiovascular Section, Department of Clinical Sciences and Community Health, University of Milan, 20159 Milan, Italy

**Keywords:** acute myocardial infarction, chronic dialysis, percutaneous coronary intervention, mortality, administrative database

## Abstract

**Background:**

Patients on chronic dialysis are less likely to be treated with percutaneous coronary intervention (PCI) for acute myocardial infarction (AMI). This is due to the lack of evidence from randomized trials, concerns about possible PCI-related side effects, and multimorbidity. Therefore, routine use of PCI for treatment of dialysis patients with AMI remains an unresolved issue.

**Methods:**

We analyzed data of patients on chronic dialysis hospitalized with AMI from 2003 to 2018, by using the administrative Lombardy Health Database (Italy). Patients were grouped according to whether they underwent or not PCI during index hospitalization. The primary outcome was in-hospital mortality, 1-year mortality was the secondary endpoint.

**Results:**

During the study period, 265,048 patients were hospitalized with AMI. Of them, 3206 (1.2%) were on chronic dialysis (age 71 ± 11; 72% males). Among dialysis patients, 44% underwent PCI, while 54% underwent PCI among non-dialysis patients (*p <* 0.0001). Dialysis was an independent predictor of treatment with medical therapy only (OR 0.75 [95% CI 0.70–0.81]). In-hospital mortality in the dialysis cohort was 15%, significantly lower in patients treated with PCI than in those not treated with PCI (11% vs. 19%; *p <* 0.0001). One-year mortality was 47% and it was lower in PCI-treated patients (33% vs. 52%; *p <* 0.0001). The adjusted risk of the study endpoints was significantly lower in dialysis patients undergoing PCI: OR 0.62 (95% CI 0.50–0.76) for in-hospital mortality; HR 0.63 (95% CI 0.56–0.71) for 1-year mortality.

**Conclusions:**

This study showed that in AMI patients on chronic dialysis, PCI is associated with a significant in-hospital and 1-year survival benefit. Yet, they underwent PCI less frequently than patients with preserved renal function.

## 1. Introduction

The cornerstone of treatment of acute myocardial infarction (AMI) is percutaneous coronary intervention (PCI), as a primary revascularization strategy in ST-segment elevation myocardial infarction (STEMI) patients and as an urgent invasive approach in those with non-ST-elevation myocardial infarction (NSTEMI) [1, 2]. In both clinical settings, PCI has been associated with a substantial decrease in hospital and long-term mortality [1, 2].

Acute myocardial infarction remains the major cause of morbidity and mortality in patients on chronic dialysis [3, 4]. However, these patients are less likely to receive PCI for AMI treatment compared to those not on dialysis [5, 6]. Indeed, they have been systematically excluded from pivotal AMI trials, leading to a lack of robust evidence whether they benefit from PCI [1, 2, 7]. In chronic dialysis patients, concerns about possible PCI-related side effects, in particular bleeding and vascular complications, multimorbidity, and insufficient safety and efficacy data for the use of anti-thrombotic therapy, complicate the AMI therapeutic decision-making process [8, 9, 10, 11]. Notably, in some registries, no impact on mortality or, even, a higher mortality risk has been reported when dialysis patients with AMI are treated with PCI [5, 6, 12, 13, 14, 15]. Thus, routine use of PCI for treatment of dialysis patients hospitalized with AMI still remains an unresolved issue despite the progressive increase of availability of PCI centers [1, 2].

We here analyzed administrative data from Lombardy, the most populous Italian region, to evaluate the rate of PCI use and its impact on in-hospital and 1-year mortality in patients on chronic dialysis hospitalized with AMI.

## 2. Methods

**Data source**. Our study used administrative health databases of the Lombardy region (Italy), which include a population registry with demographic data of all residents and detailed information on hospital records and drug prescriptions. Data are available for about 10 million registered inhabitants of Lombardy from 2000 to 2019. Healthcare in Italy is publicly funded for all residents, irrespective of social class or employment, and everyone is assigned a personal identification number kept in the National Civil Registration System. All registered residents are assisted by general practitioners and are covered by the National Health System (NHS). The pharmacy prescription database contains the medication name and anatomic therapeutic chemical classification code (ATC) and date of dispensation of drugs reimbursed by the NHS. The hospital database contains information on date of admission, discharge, death, primary diagnosis, and up to five co-existing clinical conditions and procedures performed. The diagnoses, uniformly coded according to the 9th International Code of Diseases (ICD-9-CM, International Classification of Diseases, 9th revision-Clinical Modification) and standardized in all Italian hospitals, are compiled by the hospital specialists directly in charge of the patients and are validated by hospitals against detailed clinical-instrumental data. A unique identification code allows linkage of all databases. In Italy, studies using retrospective anonymous data from administrative databases that do not involve direct access by investigators to identification data do not require Ethics Committee approval or notification nor patient informed consent signing.

**Study population**. Patients on chronic dialysis for at least 6 months and with a hospitalization due to AMI (both STEMI and NSTEMI [ICD-9-CM codes 410.x]) from 2003, through 2018, were included in the analyses. Patients undergoing coronary bypass surgery during AMI hospitalization were excluded (n = 58). Only hospitalizations in which AMI-associated ICD-9 code was listed as a primary diagnosis were abstracted. When patients were transferred between hospitals, we evaluated the complete episode of care. Patients were grouped according to whether they were treated or not with PCI during index hospitalization. Since medical information was recorded in the Lombardy registry from January 2000, past medical history was available in all patients within at least 3 years before admission. Data collection was achieved by trained reviewers.

**Study endpoints and follow-up**. The primary endpoint was in-hospital mortality. One-year all-cause mortality was considered as secondary endpoint. Patients were followed-up from the index date until death, migration or up to the end of one-year follow-up.

**Statistical analysis**. Baseline characteristics were evaluated using descriptive statistics. Categorical variables were described using frequencies and percentages and compared using Chi-square test; continuous variables were described using mean and standard deviation (SD) and compared using Student’s *t*-test.

To assess whether dialysis was an independent predictor of medical treatment only (without PCI), we applied a multivariable logistic model by considering the overall AMI cohort. This model was adjusted for all variables found to be significantly different between patients not on chronic dialysis and those on chronic dialysis. A multivariate logistic model was also used to investigate factors associated with the decision not to perform PCI in the cohort of dialysis patients. This model was adjusted for all variables found to be significantly different between patients who underwent PCI and those who did not undergo PCI.

The association between in-hospital mortality and PCI use in the dialysis cohort only was analyzed by logistic model and the results were reported as odds ratios (OR) and 95% confidence intervals (CI). The association between 1-year mortality and PCI use in the dialysis cohort only was investigated by applying Cox regression and the results were shown as hazard ratio (HR) and 95% CI. These models were adjusted for all variables found to be significantly different between patients on chronic dialysis and treated with or not treated with PCI. These analyses were performed in the whole dialysis cohort and in STEMI and NSTEMI patients, considered separately. Differences in cumulative incidence of 1-year mortality were plotted using Kaplan-Meier curves according to PCI use among patients on chronic dialysis. Moreover, a subgroup analysis on in-hospital and 1-year mortality was performed in patients on chronic dialysis not treated with PCI comparing those undergoing or not coronary angiography during hospitalization. The models were adjusted for all variables found to be significantly different between the subgroups.

The temporal trends of PCI use and in-hospital mortality in the dialysis cohort across years were assessed by Mantel-Haenszel χ^2^-test.

A two-sided *p*-value less than 0.05 was required for statistical significance. All analyses were performed using SAS version 9.4 (SAS Institute, Cary, NC, USA).

## 3. Results

**Fig. 1.**
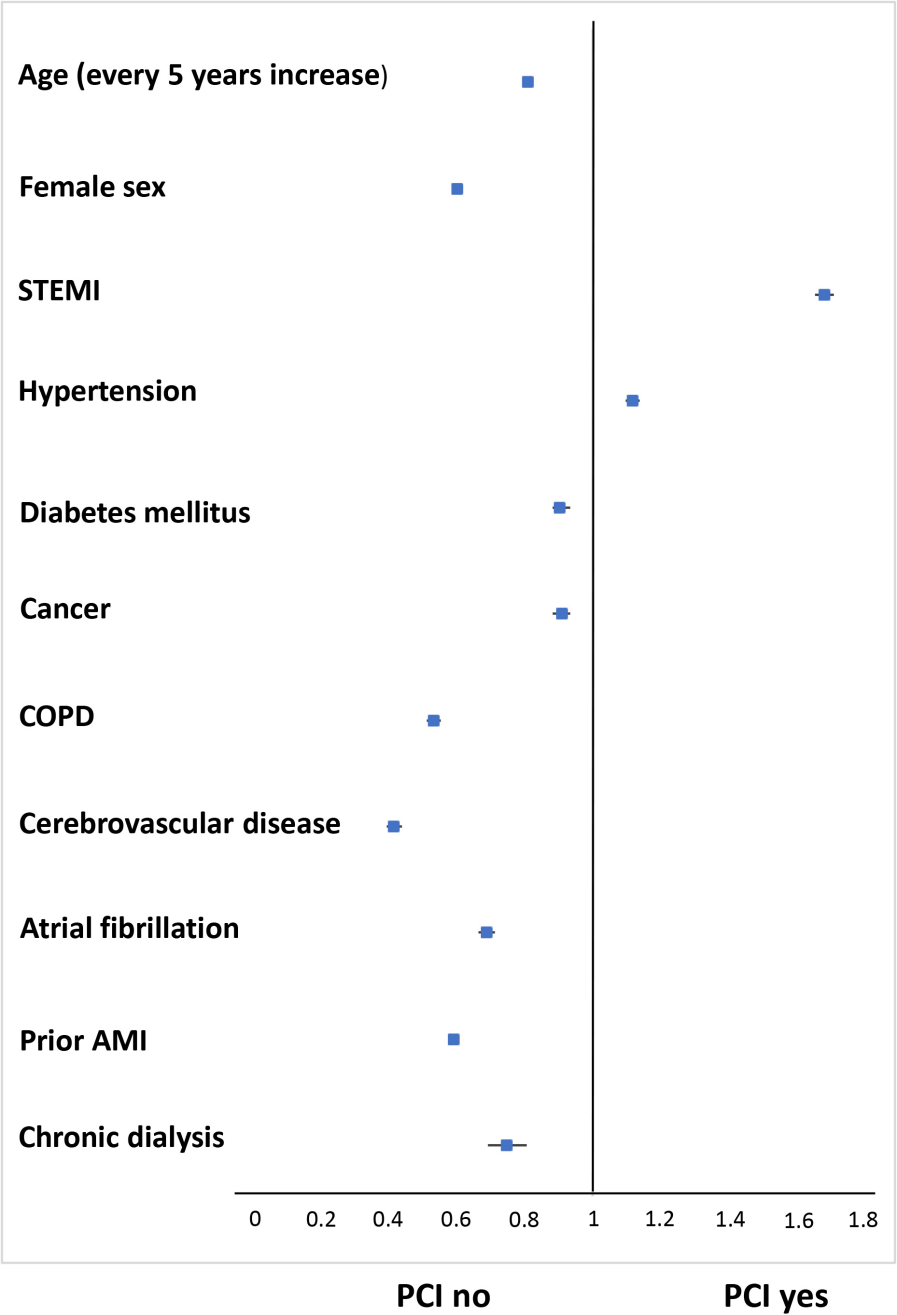
**Independent predictors of percutaneous coronary intervention (PCI) use during index hospitalization in the overall acute myocardial infarction population (n = 265,048)**. AMI, acute myocardial infarction; COPD, chronic obstructive pulmonary disease; PCI, percutaneous coronary intervention; STEMI, ST-elevation myocardial infarction.

During the considered study period, 265,048 patients hospitalized with a primary diagnosis of AMI were identified. Of them, 3206 (1.2%) on chronic dialysis (age 71 ± 11 years, 72% men, 63% with NSTEMI) were included in the study. Baseline clinical characteristics, cardiovascular medications taken before admission, and in-hospital and 1-year mortality rates in the overall AMI population and in patients under chronic dialysis or not are reported in Table 1. Patients on chronic dialysis were less frequently treated with PCI during index AMI hospitalization as compared to those not on chronic dialysis (44% vs. 54%; *p <* 0.0001). As expected, AMI patients on chronic dialysis had significantly higher in-hospital (15% vs. 6%; *p <* 0.0001) and 1-year (47% vs. 22%; *p <* 0.0001) mortality rates than those not on dialysis. In the entire study population, dialysis was an independent predictor of conservative treatment with medical therapy only, even after adjustment for all other predictors (adjusted OR 0.75 [95% CI 0.70–0.81]; Fig. 1).

**Table 1.** **Baseline characteristics of all patients hospitalized with AMI from 2003 to 2018**.

		Overall study population (n = 265,048)	Patients on chronic dialysis (n = 3206)	Patients not on chronic dialysis (n = 261,842)	*p* value
Variables					
Age (years)		71 ± 13	71 ± 11	71 ± 13	0.38
Age groups (years), n (%)					<0.0001
	<50	21,925 (8%)	163 (5%)	21,762 (8%)	
	51–60	41,301 (16%)	415 (13%)	40,886 (16%)	
	61–70	58,271 (22%)	875 (27%)	57,396 (22%)	
	71–80	72,192 (27%)	1216 (38%)	70,976 (27%)	
	>80	71,359 (27%)	537 (17%)	70,822 (27%)	
Male gender, n (%)		171,258 (65%)	2302 (72%)	168,956 (65%)	<0.0001
STEMI, n (%)		139,502 (53%)	664 (37%)	138,300 (53%)	<0.0001
PCI, n (%)		141,906 (54%)	1401 (44%)	140,505 (54%)	<0.0001
History of comorbidities, n (%) (in the previous 3 years)					
	Hypertension	83,282 (31%)	1501 (47%)	81,781 (31%)	<0.0001
	Diabetes mellitus	66,559 (25%)	1433 (45%)	65,126 (25%)	<0.0001
	Chronic IHD	58,608 (22%)	1210 (38%)	57,398 (22%)	<0.0001
	Prior AMI	26,806 (10%)	804 (25%)	26,002 (10%)	<0.0001
	Prior hospitalization for heart failure	11,913 (4.5%)	131 (4.1%)	11,782 (4.5%)	0.26
	Atrial fibrillation	15,993 (6%)	394 (12%)	15,599 (6%)	<0.0001
	COPD	14,098 (5%)	309 (10%)	13,789 (5%)	<0.0001
	Cancer	24,298 (9%)	351 (11%)	23,947 (9%)	0.0004
	Cerebrovascular disease	7447 (3%)	68 (2%)	7376 (3%)	0.018
Number of comorbidities n (%)					<0.0001
	0	104,750 (40%)	72 (2%)	104,678(40%)	
	1	82,623 (31%)	537 (17%)	82,086 (31%)	
	2	46,915 (18%)	975 (30%)	45,940 (18%)	
	3	20,607 (8%)	931 (29%)	19,676 (8%)	
	>3	10,153 (4%)	691 (22%)	9462 (4%)	
Medications of interest (before index AMI)					
	ACEi/ARB	138,702 (52%)	1654 (52%)	137,048(52%)	0.40
	Beta blockers	84,893 (32%)	1953 (61%)	82,940 (32%)	<0.0001
	Diuretics	63,144 (24%)	1719 (54%)	61,425 (23%)	<0.0001
	Calcium antagonists	76,199 (29%)	2100 (65%)	74,099 (28%)	<0.0001
	Lipid lowering drugs	91,845 (35%)	1931 (60%)	89,914 (34%)	<0.0001
	Antiplatelet drugs	117,119 (44%)	2466 (77%)	114,653 (44%)	<0.0001
	Oral anticoagulant drugs	15,069 (6%)	446 (14%)	14,623 (6%)	<0.0001
	Antihyperglycemic drugs	58,455 (22%)	1143 (36%)	57,312 (22%)	<0.0001
Endpoints					
	In-hospital mortality, n (%)	17,733 (6%)	502 (15%)	17,231 (6%)	<0.0001
	1-year mortality, n (%)	62,999 (22%)	1589 (47%)	64,410 (22%)	<0.0001

**Abbreviations**: ACEi, angiotensin-converting enzyme inhibitors; AMI, acute myocardial infarction; ARB, angiotensin receptor blockers; COPD, chronic obstructive pulmonary disease; IHD, ischemic heart disease; PCI, percutaneous coronary intervention; STEMI, ST-elevation myocardial infarction.

Table 2 shows baseline clinical characteristics and cardiovascular medications taken before admission in patients on chronic dialysis grouped according to PCI use (yes vs. no). Patients on dialysis treated with PCI were younger, more frequently males, and tended to have a less burden of comorbidities compared to those not treated with PCI. In the cohort of dialysis patients, the following were the independent predictors of the decision not to perform PCI: age (OR 1.18 [95% CI 1.14–1.22] for every 5 year increase); female gender (OR 1.37 [95% CI 1.16–1.61]); atrial fibrillation (OR 1.29 [95% CI 1.03–1.62]); chronic obstructive pulmonary disease (OR 1.40 [95% CI 1.09–1.80]); prior cerebrovascular disease (OR 2.09 [95% CI 1.19–3.67]).

**Table 2.** **Baseline characteristics of patients on chronic dialysis hospitalized with acute myocardial infarction from 2003 to 2018, grouped according to percutaneous coronary intervention use (yes vs. no)**.

		PCI yes (n = 1401)	PCI no (n = 1805)	*p* value
Variables				
Age (years)		68 ± 11	72 ± 10	<0.0001
Age groups (years), n (%)				<0.0001
	<50	100 (7%)	63 (3%)	
	51–60	233 (17%)	182 (10%)	
	61–70	429 (31%)	446 (25%)	
	71–80	457 (33%)	759 (42%)	
	>80	182 (13%)	355 (20%)	
Male gender, n (%)		1058 (76%)	1244 (69%)	<0.0001
STEMI, n (%)		538 (38%)	664 (37%)	0.35
History of comorbidities, n (%) (in the previous 3 years)				
	Hypertension	671 (48%)	830 (46%)	0.28
	Diabetes mellitus	598 (43%)	835 (46%)	0.04
	Chronic IHD	534 (38%)	676 (37%)	0.70
	Prior AMI	324 (23%)	480 (27%)	0.02
	Prior hospitalization for heart failure	50 (3%)	81 (4%)	0.19
	Atrial fibrillation	140 (10%)	254 (14%)	0.0005
	COPD	107 (8%)	202 (11%)	0.0007
	Cancer	138 (10%)	213 (12%)	0.08
	Cerebrovascular disease	17 (1%)	51 (3%)	0.002
Number of comorbidities n (%)				0.04
	0	29 (2%)	43 (2%)	
	1	236 (17%)	301 (17%)	
	2	462 (33%)	513 (28%)	
	3	399 (28%)	532 (29%)	
	>3	275 (20%)	416 (23%)	
Medications of interest (before index AMI)				
	ACEi/ARB	718 (51%)	936 (52%)	0.73
	Beta blockers	921 (66%)	1032 (57%)	<0.0001
	Diuretics	752 (54%)	967 (54%)	0.95
	Ca-antagonists	935 (67%)	1165 (65%)	0.19
	Lipid lowering drugs	873 (62%)	1058 (59%)	0.03
	Antiplatelet drugs	1100 (79%)	1366 (76%)	0.06
	Oral anticoagulant drugs	188 (13%)	258 (14%)	0.47
	Antihyperglycemic drugs	464 (33%)	679 (38%)	0.008

**Abbreviations**: ACEi, angiotensin-converting enzyme inhibitors; AMI, acute myocardial infarction; ARB, angiotensin receptor blockers; COPD, chronic obstructive pulmonary disease; IHD, ischemic heart disease; PCI, percutaneous coronary intervention; STEMI, ST-elevation myocardial infarction.

**Fig. 2.**
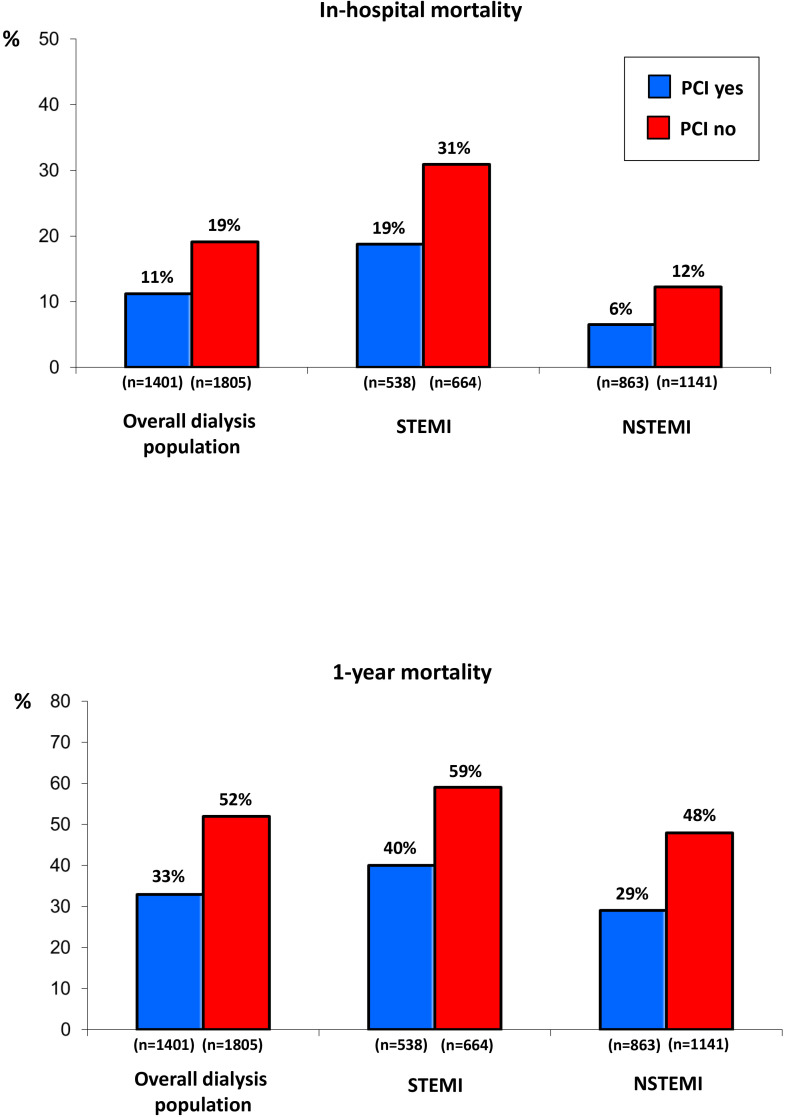
**Primary (upper panel) and secondary (lower panel) endpoint rates in the chronic dialysis population and in STEMI and NSTEMI patients considered separately, grouped according to percutaneous coronary intervention (PCI) use**. *p* < 0.001 for all comparisons. STEMI, ST-elevation myocardial infarction; NSTEMI, non-ST-elevation myocardial infarction.

**Fig. 3.**
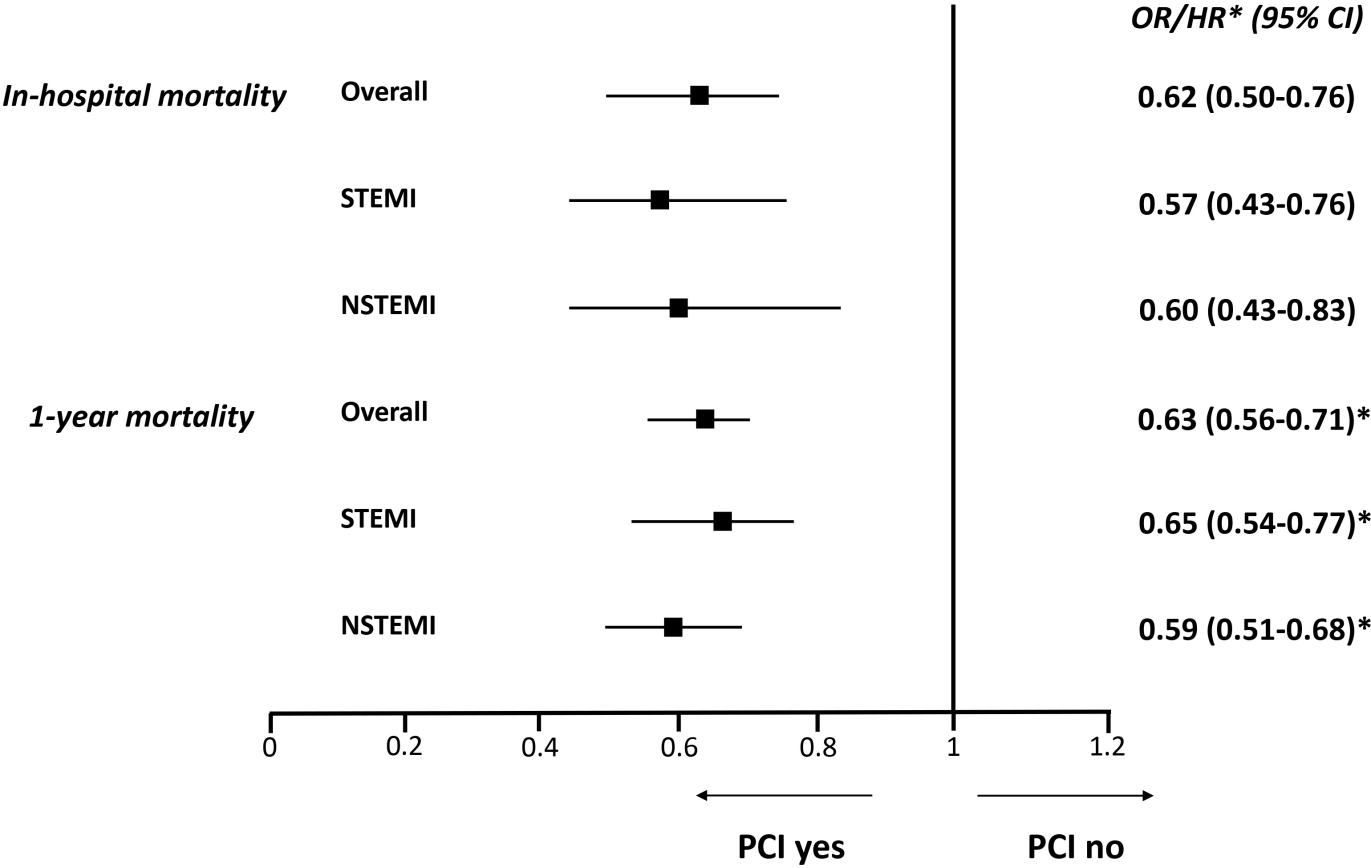
**Adjusted risk of the primary and secondary endpoints associated with percutaneous coronary intervention (PCI) use in the overall chronic dialysis population and in STEMI and NSTEMI patients considered separately**. STEMI, ST-elevation myocardial infarction; NSTEMI, non-ST-elevation myocardial infarction; CI, confidence interval; HR, hazard ratio; OR, odds ratio. “*” identifies HRs.

**Fig. 4.**
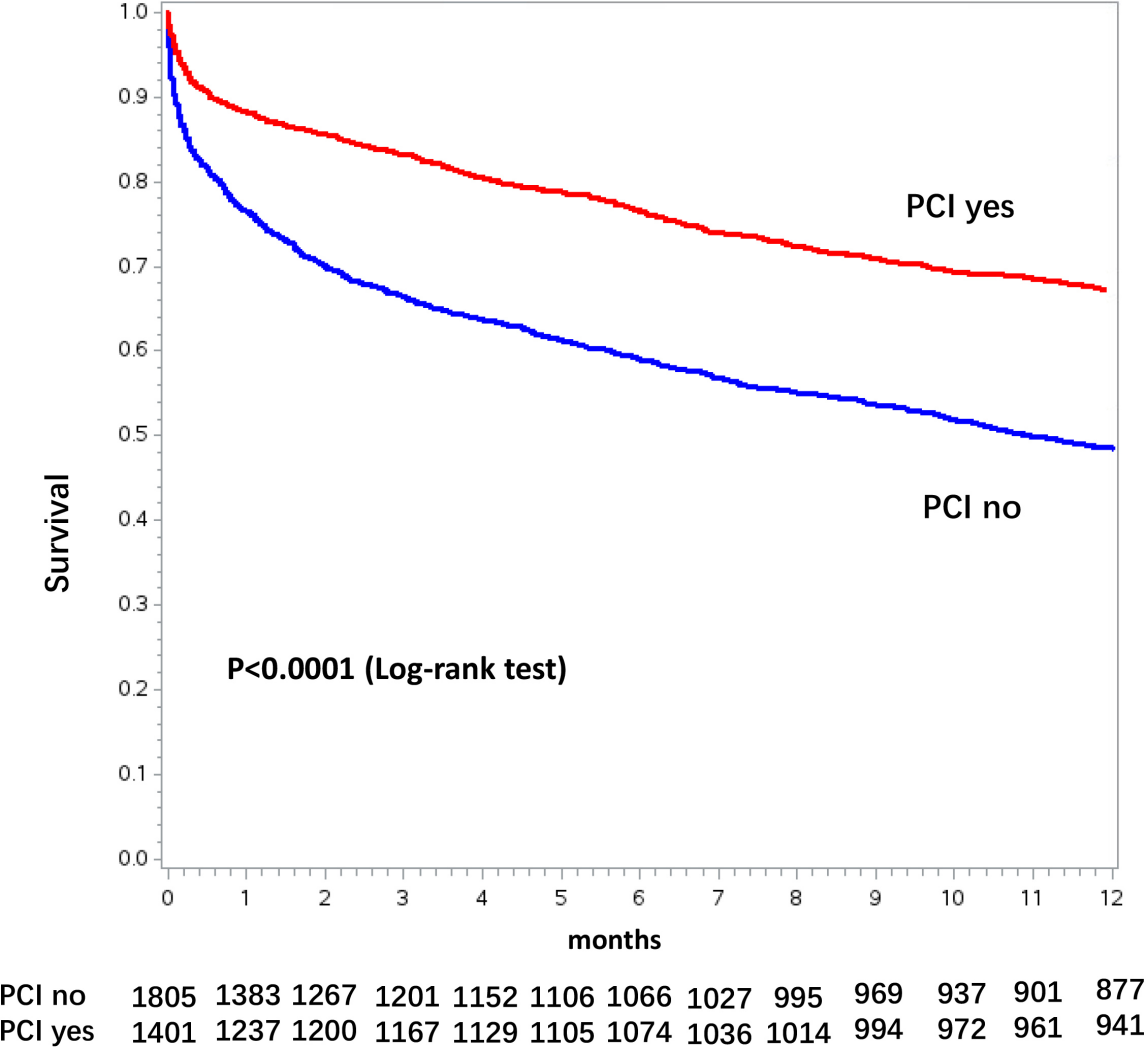
**Kaplan-Meier curves for 1-year mortality stratified by percutaneous coronary intervention (PCI) use in the overall chronic dialysis population**.

**Fig. 5.**
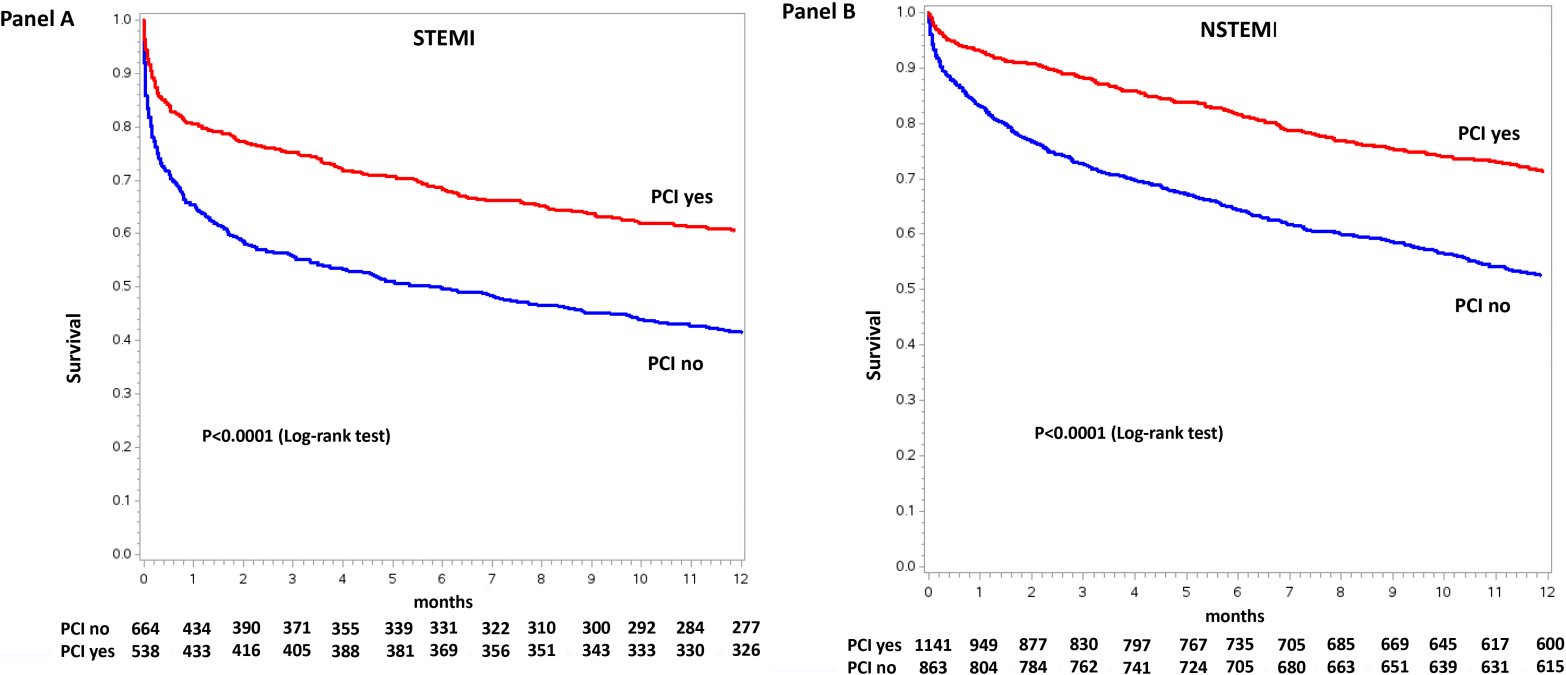
**Kaplan-Meier curves for 1-year mortality stratified by percutaneous coronary intervention (PCI) use in STEMI (Panel A) and in NSTEMI (Panel B) dialysis patients**. STEMI, ST-elevation myocardial infarction; NSTEMI, non-ST-elevation myocardial infarction.

The in-hospital mortality rate in the dialysis cohort hospitalized with STEMI was 25%, while that in patients hospitalized with NSTEMI was 10% (*p <* 0.0001). One-year mortality was 50.2% in STEMI and 40% in NSTEMI patients (*p <* 0.0001) on chronic dialysis. The rates of the primary and secondary endpoints in the chronic dialysis cohort, considered overall and grouped according to AMI type (STEMI and NSTEMI), were significantly lower in patients treated with PCI as compared to those not treated with PCI (Fig. 2). Similarly, PCI use was associated with a lower adjusted risk of both in-hospital and 1-year mortality in the overall dialysis cohort and in STEMI and NSTEMI patients (Fig. 3). The Kaplan-Meyer curve for 1-year mortality in dialysis patients treated or not with PCI during index hospitalization is shown in Fig. 4. The Kaplan-Meyer curves for 1-year mortality in dialysis patients treated or not with PCI stratified according to AMI type (STEMI or NSTEMI) are shown in Fig. 5.

Among patients on chronic dialysis not treated with PCI, 555 (31%) underwent diagnostic coronary angiography. They were older, more frequently presented with STEMI and had a higher prevalence of chronic IHD, atrial fibrillation and chronic obstructive pulmonary disease (COPD) than patients not treated with PCI (**Supplementary Table 1**). After adjustment for possible confounding factors, patients undergoing coronary angiography and treated conservatively experienced a significantly lower risk of in-hospital (OR 0.55 [95% CI 0.41–0.75]) and 1-year (HR 0.70 [95% CI 0.60–0.81]) mortality.

**Fig. 6.**
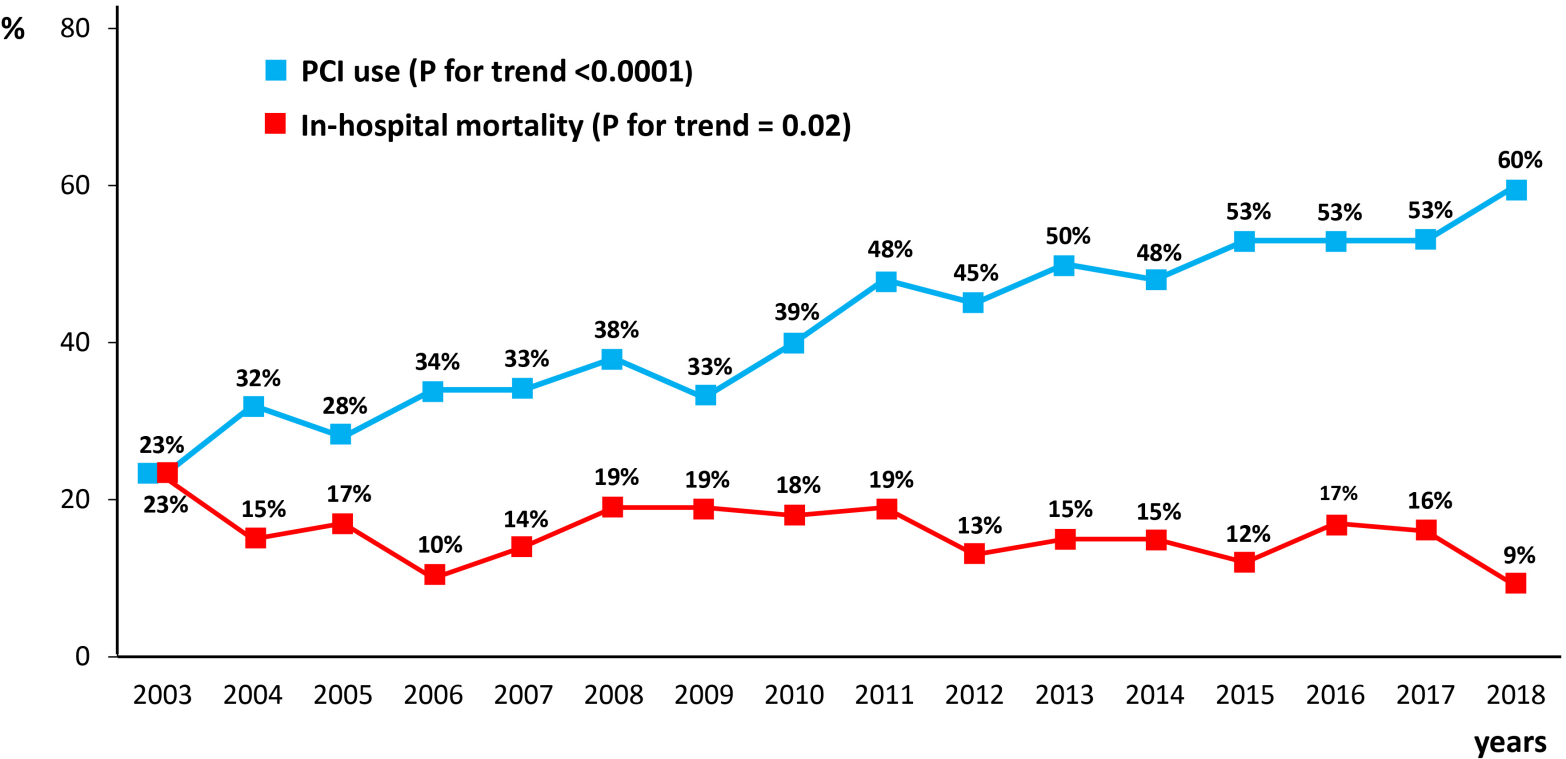
**Rates of in-hospital mortality and percutaneous coronary intervention (PCI) use in the overall chronic dialysis population over the study period (2003–2018)**.

Fig. 6 reports the temporal trend of PCI use and of in-hospital mortality during the considered study period in AMI patients on chronic dialysis. The use of PCI progressively increased over time, from 23% in 2003 to 60% in 2018 (*p* for trend <0.0001). Conversely, in-hospital mortality decreased from 23% in 2003 to 9% in 2018 (*p* for trend = 0.02).

## 4. Discussion

Cardiovascular disease is the leading cause of morbidity and mortality in patients on chronic dialysis. Indeed, although traditional coronary risk factors are frequent in the dialysis population [16, 17, 18], these patients are also exposed to other non-traditional uremia-specific cardiovascular risk factors, including inflammation, increased oxidative stress, and neuro-hormonal activity [19]. Moreover, when dialysis patients are hospitalized with AMI, their mortality is significantly higher than that of non-dialysis patients, with reported in-hospital mortality rates of 10%–30% and 1-year and 2-year mortality rates as high as 30% and 60%, respectively [5, 6, 12]. Thus, despite patients on chronic dialysis represent only a small proportion (0.5%–1.5%) of all patients hospitalized with AMI, they still have a prohibitive mortality risk [5, 6].

The poor outcome of dialysis patients with AMI can be explained not only by delay in the diagnosis, atypical symptoms, and higher burden of comorbid conditions but also by some therapeutic nihilism [5, 6, 7, 8, 9, 10, 11, 12, 13, 14, 15]. In particular, dialysis patients are more likely to be treated conservatively, without PCI, than their counterparts [5, 6]. This is due to lack of data from major clinical trials of cardiovascular interventions that have systematically excluded them [7], concerns about the long-term safety and efficacy of antiplatelet therapy, increased procedural bleeding risk, and technical challenge of intervening in calcified coronary vessels [8, 9, 10, 11]. Finally, some registries have reported no impact on mortality or, even, a higher mortality risk when dialysis patients with AMI are treated with PCI [5, 6, 12, 13, 14, 15]. For example, the Swedish Web-System for Enhancement and Development of Evidence-Based Care in Heart Disease Evaluated according to Recommended Therapies (SWEDEHEART) registry concluded that there is no survival benefit, both during hospitalization and at 1-year follow-up, of invasive strategies in dialysis patients with NSTEMI [5]. Accordingly, a systematic review did not recently support an early invasive treatment of NSTEMI in dialysis patients [20]. Similarly, no advantage of primary PCI over thrombolysis or medical therapy only for treatment of STEMI in patients with severe chronic kidney disease or dialysis was reported in the Global Registry of Acute Coronary Events (GRACE) registry [21]. Thus, PCI is still underused in dialysis patients with AMI and its impact on mortality remains unclear for this high-risk and highly vulnerable population.

On these bases, we analyzed a large administrative real-world dataset to investigate the clinical impact of PCI in dialysis patients hospitalized with AMI. We found that dialysis patients represent about 1% of all AMI patients hospitalized in Lombardy between 2003 and 2018, with an overall in-hospital mortality of 15% and a 1-year mortality of 47%. These prevalence and mortality rates are similar to those reported in other studies [5, 6, 20, 21]. In particular, chronic dialysis patients constituted approximately 1% of all hospital admissions in the GRACE population, with an-in-hospital mortality of 13% [6]. In the SWEDEHEART registry, 1-year mortality was 51% [5].

In our study, patients on chronic dialysis hospitalized with AMI were less likely undergo PCI compared to non-dialysis patients, even after considering all major confounders for PCI referral. Notably, PCI was performed in only 44% of the dialysis population. However, when dialysis patients were treated with PCI, regardless of AMI type (STEMI or NSTEMI), their in-hospital and 1-year mortality was significantly lower than those of patients not treated with PCI. In particular, the adjusted risk of in-hospital mortality and that of 1-year mortality were reduced by almost 40% in the overall dialysis cohort. A similar prognostic benefit associated with PCI was observed in STEMI and NSTEMI patients considered separately. Notably, among patients not treated with PCI, those undergoing diagnostic coronary angiography experienced a lower in-hospital and 1-year mortality than those not undergoing coronary angiography. This suggests that coronary angiography during AMI hospitalization, regardless of PCI use, is a useful prognostic stratification tool also in patients on chronic dialysis, allowing to identify the more appropriate therapeutic strategy (medical therapy versus PCI). Therefore, in contrast with recent clinical trials performed in chronic coronary disease populations, that have not demonstrated the superiority of PCI added to medical therapy over a strategy of medical therapy alone [22, 23, 24], both in patients with advanced kidney disease and in patients without, our data support the advantage of PCI in the specific context of AMI even in presence of chronic dialysis treatment.

We also evaluated whether the use of PCI in AMI patients on chronic dialysis changed over the considered time frame of 15 years and whether this was associated with a change in hospital mortality. As PCI use increased from 23% to 60% across the study period while in-hospital mortality decreased from 23% to 9%, it can be speculated that increased use of PCI contributes, at least in part, to the gradual reduction in mortality. This is in line with the study by Shroff *et al*. [25], which reported a decline in hospital and 2-year mortality among dialysis patients with AMI in the United States between 1993 and 2008, coincident with increased in-hospital PCI rates. Although there are several aspects that remain to be investigated, including PCI-related complications, our data strongly support that chronic dialysis per se should not be considered a decisive factor in precluding coronary angiography/PCI to patients hospitalized with AMI.

Administrative databases allow to investigate outcomes of large cohorts representing the real clinical care setting, as they collect data over time in a standardized fashion [26, 27]. In particular, they are valuable for examining real-world practice patterns among populations not well-represented, or even excluded, in randomized clinical trials or registries. However, limitations typical of administrative datasets need to be acknowledged. First, administrative data can suffer from systematic biases as their quality depends on coding accuracy. In particular, biases may have resulted from underreporting or changes in AMI diagnosis or coding patterns over time. Yet, it should be highlighted that the endpoints considered in our study, in particular in-hospital and 1-year mortality, are less likely to be subject to coding error. Second, some specific pieces of information on clinical variables or laboratory tests closely associated with the study endpoints, in particular left ventricular ejection fraction, acute pharmacologic therapy, completeness of myocardial revascularization, and other known risk factors were not available [28]. Third, the impact of 1-year mortality of discharge therapy was not evaluated. Fourth, our study included patients from multiple hospitals over many years, and there is potential for temporal variation in care practices. Fifth, the generalizability of our findings to other countries may be limited. Finally, PCI use was not assigned randomly which could lead to possible selection bias despite adjustments made in the statistical model. Indeed, we cannot exclude that dialysis patients treated with PCI may have been healthier or hospitalized earlier than those treated medically, leading to an overestimation of the potential benefit of PCI.

## 5. Conclusions

Our study shows that patients on chronic dialysis hospitalized with AMI have an associated lower in-hospital and 1-year mortality when undergoing PCI but are less likely to be treated, which may explain, at least part, their worse outcome. The progressive increase in the use of PCI over the years may partially account for the reduction in the overall mortality of this high-risk subset of patients.

## Data Availability

Access to data is allowed within the agreement between the Centro Cardiologico Monzino, I.R.C.C.S, Milan, Italy and Regional Health Ministry of Lombardy. Thus, the data underlying this article were provided by Regional Health Ministry of Lombardy and will be shared on request to the corresponding author with permission of Regional Health Ministry of Lombardy.

## Supplementary Material

Supplementary material associated with this article can be found, in the online version, at https://doi.org/10.31083/j.rcm2405135.
