# Supplementary material for: Prognostic Impact of Percutaneous Coronary Intervention in Chronic Dialysis Patients with Acute Myocardial Infarction: Findings from the Lombardy Health Database
Source: Rev Cardiovasc Med. 2023 Apr 28;24(5):135. doi: 10.31083/j.rcm2405135 (PMC11273045; doi:10.31083/j.rcm2405135)
Supplement: Supplementary file 1 [file 2153-8174-24-5-135-s1.zip › 2153-8174-24-5-135-s1.docx]

**Supplementary Table 1.** Baseline characteristics of chronic dialysis patients hospitalized with acute myocardial infarction from 2003 to 2018 and treated conservatively.

|  | **Patients undergoing coronary angiography** (n=555) | **Patients not undergoing coronary angiography** (n=1,250) | **P value** |
| --- | --- | --- | --- |
| **Variables** |  |  |  |
| **Age** (years)**,**  mean ± SD | 70±10 | 73±10 | <.0001 |
| **Age groups** (years), n (%) |  |  |  |
| <50 | 27 (5%) | 36 (3%) | <.0001 |
| 51-60 | 76 (14%) | 106 (8%) |  |
| 61-70 | 171 (31%) | 275 (22%) |  |
| 71-80 | 227 (41%) | 532 (43%) |  |
| >80 | 54 (10%) | 301 (24%) |  |
| **Gender** (female) | 161 (29%) | 400 (32%) | 0.21 |
| **STEMI, n (%)** | 178 (32%) | 486 (39%) | 0.006 |
| **History of comorbidities,** n (%) (in the previous 2 years) |  |  |  |
| Hypertension | 257 (46%) | 573 (46%) | 0.85 |
| Diabetes mellitus | 246 (44%) | 589 (47%) | 0.27 |
| Chronic IHD | 187 (34%) | 489 (39%) | 0.03 |
| Prior hospitalization for HF | 22 (4%) | 59 (5%) | 0.47 |
| Atrial fibrillation | 58 (10%) | 196 (16%) | 0.003 |
| COPD | 46 (8%) | 156 (12%) | 0.009 |
| Cancer | 58 (10%) | 155 (12%) | 0.24 |
| Cerebrovascular disease | 14 (3%) | 37 (3%) | 0.60 |
| **Number of comorbidities**  n (%) |  |  |  |
| 0 | 19 (3%) | 24 (2%) | 0.05 |
| 1 | 100 (18%) | 201 (16%) |  |
| 2 | 167 (30%) | 346 (28%) |  |
| 3 | 160 (29%) | 372 (30%) |  |
| >3 | 109 (20%) | 307 (25%) |  |
| **Medications of interest**  (before index hospitalization) |  |  |  |
| ACEi/ARB | 289 (52%) | 647 (52%) | 0.90 |
| Beta blockers | 315 (57%) | 717 (57%) | 0.81 |
| Diuretics | 264 (48%) | 703 (56%) | 0.0007 |
| Ca-antagonists | 361 (65%) | 804 (64%) | 0.77 |
| Lipid lowering drugs | 341 (61%) | 717 (57%) | 0.10 |
| Antiplatelet drugs | 418 (75%) | 948 (764%) | 0.81 |
| Oral anticoagulant drugs | 73 (13%) | 185 (150%) | 0.36 |
| Antihyperglycemic drugs | 190 (34%) | 489 (39%) | 0.05 |
| **In-hospital mortality, n (%)** | 67 (12%) | 278 (22%) | <0.0001 |
| **1-year mortality , n (%)** | 222 (40%) | 713 (57%) | <0.0001 |

**Abbreviations**: ACEi=angiotensin-converting enzyme inhibitors; ARB=angiotensin receptor blockers; COPD=chronic obstructive pulmonary disease; HF= heart failure; IHD=ischemic heart disease; PCI=percutaneous coronary intervention; STEMI=ST-elevation myocardial infarction.
